# Supplementary material for: Low HDL-cholesterol levels predict hepatocellular carcinoma development in individuals with liver fibrosis
Source: JHEP Rep. 2022 Nov 15;5(1):100627. doi: 10.1016/j.jhepr.2022.100627 (PMC9763866; doi:10.1016/j.jhepr.2022.100627)
Supplement: Multimedia component 1 [file mmc1.pdf]

Journal of Hepatology, Volume *5*

Supplemental information

Low HDL-cholesterol levels predict hepatocellular carcinoma development in individuals with liver fibrosis

Lucilla Crudele, Carlo De Matteis, Elena Piccinin, Raffaella Maria Gadaleta, Marica Cariello, Ersilia Di Buduo, Giuseppina Piazzolla, Patrizia Suppressa, Elsa Berardi, Carlo Sabbà, and Antonio Moschetta

Low HDL-cholesterol levels predict hepatocellular carcinoma development in individuals with liver fibrosis

Lucilla Crudele, Carlo De Matteis, Elena Piccinin, Raffaella Maria Gadaleta, Marica Cariello, Ersilia Di Buduo, Giuseppina Piazzolla, Patrizia Suppressa, Elsa Berardi, Carlo Sabbà, Antonio Moschetta

Table of content

Table S1..2

Table S1. Child-Pugh and Meld Scores in patients who developed HCC.

Patient ID	Child-Pugh	Meld Score-Na
1	6	13
2	5	8
3	6	9
4	5	7
5	6	11
6	8	10
7	5	9
8	5	9
9	5	9
10	5	7
11	6	11
12	6	9
13	7	7
14	7	8
15	6	7
16	5	7
**Mean±SD**	**5.81±0.88**	**8.81±1.70**
