# Supplementary material for: Low HDL-cholesterol levels predict hepatocellular carcinoma development in individuals with liver fibrosis
Source: JHEP Rep. 2022 Nov 15;5(1):100627. doi: 10.1016/j.jhepr.2022.100627 (PMC9763866; doi:10.1016/j.jhepr.2022.100627)
Supplement: Multimedia component 4 [file mmc4.pdf]

Low HDL-cholesterol levels predict hepatocellular carcinoma development in individuals with liver fibrosis

Lucilla Crudele,[1,†] **Carlo De Matteis**,[1,2,†] Elena Piccinin,[3] Raffaella Maria Gadaleta,[1] Marica Cariello,[1] Ersilia Di Buduo,[1] Giuseppina Piazzolla,[1] Patrizia Suppressa,[1] Elsa Berardi,[1] Carlo Sabbà,[1] Antonio Moschetta[1,2,*]

[1]Department of Interdisciplinary Medicine, University of Bari "Aldo Moro", Piazza Giulio Cesare 11, 70124 Bari, Italy; [2]INBB National Institute for Biostructure and Biosystems, Viale delle Medaglie d'Oro 305 - 00136 Roma, Italy; [3]Department of Basic Medical Science, Neurosciences and Sense organs, University of Bari "Aldo Moro", Piazza Giulio Cesare 11, 70124 Bari, Italy

JHEP Reports **2023.** https://doi.org/10.1016/j.jhepr.2022.100627

Background & Aims: Dysmetabolic conditions could drive liver fibrosis in patients with non-alcoholic fatty liver disease (NAFLD), increasing susceptibility to hepatocellular carcinoma (HCC). We therefore aimed to identify novel predictive biomarkers of HCC in patients with and without liver fibrosis.
Methods: A total of 1,234 patients with putative metabolic conditions and NAFLD were consecutively assessed in our outpatient clinic. Clinical and biochemical data were recorded, and then liver ultrasonography was performed annually for 5 years to detect HCC onset. For the analysis, the population was first divided according to HCC diagnosis; then a further subdivision of those who did not develop HCC was performed based on the presence or absence of liver fibrosis at time 0.
Results: Sixteen HCC cases were recorded in 5 years. None of our patients had been diagnosed with cirrhosis before HCC was detected. Compared to patients who did not develop HCC, those who did had higher liver transaminases and fibrosis scores at time 0 (p <0.001). In addition, they presented with increased glycated haemoglobin levels and lower 25-OH vitamin D levels (p <0.05). Intriguingly, patients with higher liver fibrosis scores who subsequently developed HCC had lower HDL-cholesterol (HDL-c) levels at time 0 (p <0.001). Furthermore, in the 484 patients presenting with lower HDL-c at baseline, we found that waist circumference, and then vitamin D and glycated haemoglobin levels, were significantly different in those who developed HCC, regardless of liver fibrosis (p <0.05).
Conclusions: This study identifies HDL-c as a *bona fide* novel marker to predict HCC in patients with NAFLD. Increased waist circumference and deranged metabolic pathways represent additional predisposing factors among patients with low HDL-c, highlighting the importance of studying cholesterol metabolism and integrating clinical approaches with dietary regimens and a healthy lifestyle to prevent HCC.
Impact and implications: Visceral adiposity and its associated conditions, such as chronic inflammation and insulin resistance, may play a pivotal role in hepatocellular carcinoma development in patients with non-alcoholic fatty liver disease. We provide new insights on the underlying mechanisms of its pathogenesis, shedding light on the involvement of low levels of "good" HDL-cholesterol. We recommend integrating dietary regimens and advice on healthy lifestyles into the clinical management of non-alcoholic fatty liver disease, with the goal of reducing the incidence of hepatocellular carcinoma.
© 2022 The Author(s). Published by Elsevier B.V. on behalf of European Association for the Study of the Liver (EASL). This is an open access article under the CC BY license (http://creativecommons.org/licenses/by/4.0/).

Introduction

Hepatocellular carcinoma (HCC) represents the sixth most common neoplasm in terms of incidence and the third leading cause of cancer death.[1] Despite progress being made in the prevention, early detection, and diagnosis of this disease, it remains a bleak field of unmet medical needs. Since traditional therapeutic management achieves good results only in earlier stages, limiting the predisposing risk factors for HCC is likely the best strategy to decrease both its onset and associated mortality. Indeed, in the Western world, around 40% of HCC cases are mostly attributable to metabolic conditions, such as non-alcoholic fatty liver disease (NAFLD), metabolic syndrome (MetS), and type 2 diabetes.[2,3] NAFLD is characterised by the accumulation of lipids within hepatocytes; it can progress to non-alcoholic steatohepatitis (NASH) and it can be accompanied by fibrosis progression (41%).[4] If not promptly reverted, NASH can lead to cirrhosis, accounting for the most non-infectious and non-alcoholic cases of HCC.[5] However, several studies have demonstrated that liver cancer can also arise in individuals with NAFLD/NASH without cirrhosis.[6,7] Therefore, since metabolic alterations are now recognized as an important hallmark of cancer, healthy lifestyle habits may not only improve MetS and NAFLD conditions, but also have an impact on cancer incidence. Indeed, tumour cells modify their metabolism to fulfil the increasing energy demands of sustaining continuous proliferation and

Keywords: NAFLD; NASH; Metabolic syndrome; Waist circumference; Vitamin D; APRI score.
Received 23 July 2022; received in revised form 5 October 2022; accepted 3 November 2022; available online 15 November 2022
† These authors contributed equally to the work.
* Corresponding author. Address: Department of Interdisciplinary Medicine, University of Bari Aldo Moro, 70121 Bari, Italy. Tel: +39 0805593262.
E-mail address: antonio.moschetta@uniba.it (A. Moschetta).

growth.[8] In this context, dysregulated lipid metabolism is one of the most important factors to consider. Specifically, alterations in cholesterol and fatty acid (FA) metabolism are important drivers of tumour progression, and in particular HCC.[9–14] To satisfy the increasing energy demand, cancer cells can either increase *de novo* synthesis of FAs and cholesterol or promote the uptake of exogenous lipids. Thus, dietary carbohydrates that drive hepatic *de novo* lipogenesis and dietary lipids could also contribute to an increased risk of cancer development. Moreover, specific lipid classes – including saturated FAs and cholesterol – have been strongly associated with disease progression.[15]

HDL plays a crucial role in preventing atherosclerosis via the reverse cholesterol transport (RCT) pathway, through which dietary cholesterol is transported from peripheral tissues to the liver where it is converted into bile salts that can be removed from the body in faeces. HDL-cholesterol (HDL-c) also shows anti-inflammatory and antioxidant properties, which is why it is known as “good cholesterol”. Furthermore, a negative correlation between HDL-c level and diagnosis of MetS and NAFLD exists[16,17] and alterations in HDL formation and remodelling might have a direct impact on liver carcinogenesis.[18] Indeed, under the pressure of hyperinsulinemia, hyperglycaemia, and systemic lipid imbalance, hepatocytes rewire their metabolism, including HDL-related pathways, thus priming NAFLD development and its progression to chronic liver disease and HCC.[19] The potential role of HDL-c in predicting HCC risk has been intensively debated, especially in patients affected by metabolic diseases, since low HDL is one of the criteria associated with MetS and NAFLD. In the general population, low HDL-c levels are associated with increased cancer mortality rates, although the relation follows more of a J-shaped pattern rather than an inverse one, possibly due to the presence of some genetic variants that might have adverse effects on health outcomes.[20,21] Among patients with MetS, the individual diagnostic criteria of MetS were associated with a higher risk of liver cancer, with low HDL-c alone increasing the risk up to 16%.[5] Also, in a cohort of patients with diabetes, an increase of 15 mg/dl in the HDL-c values has been associated with a 9% and 6% diminished risk of cancer in men and women, respectively, this inverse association still being present after stratification of the population by race, BMI, smoking, and medication use.[22] Even if it is still unclear whether the observed association is causal or due to preclinical diseases, such as dysmetabolism or increased cholesterol influx in hepatic cells, HDL dysfunctions may represent another possible pathogenic link between MetS, NAFLD and liver cancer, in addition to insulin resistance and low-grade inflammation.

Accurate low-cost and non-invasive screening tools that predict HCC risk are a critical unmet need. Hence, and in light of the potential involvement of deranged cholesterol metabolism in cancer development, we screened 1,234 patients with NAFLD using lipid biomarker levels and the non-invasive fibrosis score APRI (aspartate aminotransferase-to-platelet ratio index) to detect additional drivers of HCC and to determine if they could be used as new predictive biomarkers of HCC development.

Patients and methods

Study participants

Patients’ enrolment, anthropometric, biochemical and clinical variables were recorded in the electronic health register of Metabolic Diseases of the Department of Interdisciplinary Medicine at the “Aldo Moro” University of Bari (Italy) from January 2017 to January 2022. First, a total of 1,545 outpatients suspected of having MetS and fatty liver were enrolled in this study. All participants underwent a physical examination, biochemical assessment and abdominal ultrasound.

Then, patients with reported alcohol abuse, viral hepatitis, benign or primary liver cancer, inflammatory bowel disease, celiac disease, acute heart diseases (cardiac failure, coronary arterial disease, acute arrhythmias), renal and hepatic failure, infections, secondary hypertension caused by renal or endocrine and neurogenic conditions, as well as aortic coarctation, chronic systemic inflammatory diseases, and neoplastic diseases with recent onset (less than 10 years) and/or under chemotherapy at baseline were excluded, leaving 1,234 patients who were included in the study.

At first ultrasonography assessment, no patients had been diagnosed with cirrhosis, consequently they were screened with liver ultrasonography every year in the following 5 years, according to our institutional screening and follow-up policy for patients with metabolic conditions. Statistical analysis was performed on a final total population of 1,234 patients (605 males, 629 females). The study was approved by the Ethics Committee of the Azienda Ospedaliero-Universitaria Policlinico di Bari (Bari, Italy) in accordance with the requirements of the Declaration of Helsinki. Written informed consent for the use of clinical data was obtained from all participants in the study. In accordance with the approved Ethics Committee, only patients who were already 18 years old or more were included.

Clinical assessment

Anthropometric assessment was performed using standardized procedures. Briefly, waist circumference (WC) was measured at the midpoint between the inferior part of the 12th rib and the anterior-superior iliac crest. BMI was computed as weight (kg) divided by the height (m) squared. Average systolic and diastolic blood pressure parameters were registered for each patient as the mean of three measurements using a manual sphygmomanometer after a period of rest of at least 15 min. Abdominal ultrasound was performed to exclude HCC at time 0 with an Esaote My Lab 70 Gold ultrasound system with 2.5–5 MHz convex probes. The cardiovascular risk (CVR) was calculated using the official Framingham Heart Study estimator for cardiovascular disease in the upcoming 10-years adjusted for lipids.

APRI and fibrosis-4 (FIB-4) were used as non-invasive liver fibrosis indexes. APRI score was calculated as aspartate aminotransferase (AST) (U/L)/platelet count ($\times 10^6$/L) × 100. The cut-offs adopted were as follows: APRI <0.5 to identify a fibrosis-free liver, APRI ≥0.5 for liver fibrosis and APRI ≥1.5 for probable cirrhosis. The FIB-4 index was calculated as age × AST (U/L)/platelet count ($\times 10^6$/L) × √ alanine aminotransferase (ALT) (U/L). The cut-offs adopted were as follows: FIB-4 <1.45 for no or moderate fibrosis 1.45≤ FIB-4 <3.25 for moderate fibrosis, FIB-4 ≥3.25 for extensive fibrosis or cirrhosis.[23] Even if no patients had previously been diagnosed with cirrhosis, Child-Pugh[24] and MELD-Na (model for end-stage liver disease-Na)[25,26] scores were also computed in patients who later developed HCC.

Biochemical measurements

To analyse biochemical markers of glucose and lipid metabolism, serum was collected after overnight fasting and was processed following standardized biochemical procedures.

Table 1. Clinical characterisation of the study population.

Clinical variables	No HCC-APRI <0.5<br>1084 M:F (498:586)<br>Mean ± SD	No HCC-APRI ≥0.5<br>134 M:F (94:40)<br>Mean ± SD	HCC-APRI ≥0.5<br>16 M:F (13:3)<br>Mean ± SD	*p* value
Weight (kg)	75.84 ± 3.22	91.66 ± 2.99	95.99 ± 5.98[a]	<0.05
BMI (kg/m^2)	27.04 ± 4.08	31.09 ± 3.21[a]	33.81 ± 5.38[a]	<0.05
Waist circumference (cm)	94.29 ± 2.88	97.88 ± 4.93	107.43 ± 5.23[a]	<0.05
Cardiovascular risk (Framingham)	16.89 ± 1.02	26.48 ± 3.29[a]	27.05 ± 6.93[a]	<0.05
Sistolic blood pressure (mmHg)	127.83 ± 3.29	130.77 ± 4.10	130.95 ± 11.39	n.s.
Total cholesterol (mg/dl)	169.28 ± 5.21	179.34 ± 6.23	183.05 ± 10.23	n.s.
HDL-c (mg/dl)	57.39 ± 4.99	51.34 ± 5.30	32.09 ± 8.83[a,b]	<0.001
LDL-c (mg/dl)	104.34 ± 5.23	114.23 ± 6.23	107.95 ± 11.32	n.s.
Triglycerides (mg/dl)	120.93 ± 6.22	146.24 ± 7.12[a]	162.52 ± 19.23[a]	<0.05
Glucose (mg/dl)	94.78 ± 4.33	101.74 ± 7.99	108.73 ± 9.47[a]	<0.05
HbA1c (mmol/mol)	33.82 ± 1.92	39.77 ± 2.49	43.72 ± 5.23[a,b]	<0.05
TSH (mUI/L)	1.74 ± 0.93	1.8 ± 0.78	1.60 ± 1.38	n.s.
FT4 (ng/dl)	1.05 ± 0.64	1.07 ± 0.83	0.94 ± 0.85	n.s.
FT3 (pg/ml)	2.85 ± 0.94	2.20 ± 0.92	3.02 ± 1.04	n.s.
25-OH vitamin D (ng/ml)	25.48 ± 2.33	21.77 ± 1.48	14.99 ± 2.99[a,b]	<0.05
Homocysteine (μmol/L)	12.42 ± 1.73	14.94 ± 1.02	14.89 ± 7.34	n.s.
Folate (ng/ml)	6.75 ± 1.32	6.23 ± 1.04	5.25 ± 2.94	n.s.
hs-CRP (mg/L)	4.74 ± 0.94	3.73 ± 2.8	3.59 ± 1.79	n.s.
ESR (mm/h)	17.05 ± 15.2	18.3 ± 17.7	16.8 ± 14.6	n.s.
Iron (μg/dl)	108.97 ± 6.43	111.97 ± 7.99	91.85 ± 15.93	n.s.
Serum ferritin (ng/ml)	131.57 ± 7.94	155.72 ± 7.93	153.09 ± 149.23	n.s.
Creatinine (mg/dl)	0.91 ± 0.32	0.93 ± 0.75	0.93 ± 0.73	n.s.
GFR (ml/min)	99.23 ± 2.43	91.77 ± 1.48[a]	86.41 ± 6.20[a]	<0.05
WBC (x10^3/μl)	6.05 ± 1.10	6.77 ± 0.84	7.33 ± 1.75	n.s.
Haemoglobin (g/dl)	14.55 ± 2.91	14.55 ± 3.62	14.06 ± 2.93	n.s.
Neutrophils (%)	56.28 ± 3.23	56.28 ± 3.21	59.81 ± 4.29	n.s.
Eosinophils (%)	2.85 ± 0.93	2.85 ± 0.84	3.67 ± 0.99	n.s.
Basophils (%)	0.58 ± 0.35	0.58 ± 0.32	0.64 ± 0.75	n.s.
Lymphocytes (%)	31.74 ± 2.14	31.74 ± 3.29	29.77 ± 6.92	n.s.
Monocytes (%)	6.60 ± 1.02	6.60 ± 1.01	9.41 ± 2.92	n.s.
Platelet count (x10^3/μl)	241.97 ± 6.55	167.88 ± 4.62[a]	187.75 ± 15.23[a,b]	<0.05
GGT (U/L)	30.40 ± 3.23	55.70 ± 5.93[a]	79.71 ± 19.83[a,b]	<0.001
AST (U/L)	20.70 ± 1.48	56.07 ± 3.29[a]	51.82 ± 4.73[a]	<0.05
ALT (U/L)	30.59 ± 1.83	49.98 ± 2.94[a]	54.24 ± 5.28[a,b]	<0.05
ALP (U/L)	48.95 ± 2.02	53.95 ± 2.04	70.76 ± 14.23[a,b]	<0.05
Insulin (μIU/ml)	10.88 ± 1.39	13.72 ± 1.89	14.27 ± 2.93	n.s.
NL ratio	1.98 ± 0.93	1.81 ± 0.84	2.48 ± 0.99	n.s.
LM ratio	5.46 ± 0.99	5.57 ± 0.84	5.79 ± 1.49	n.s.
MH ratio	8.18 ± 1.52	8.77 ± 1.34	13.89 ± 1.48[a,b]	<0.05
ML ratio	0.21 ± 0.12	0.33 ± 0.09	0.29 ± 0.39	n.s.
FIB-4 index	1.12 ± 0.99	1.88 ± 0.43[a]	2.16 ± 0.56[a]	<0.001
APRI score	0.31 ± 0.08	0.62 ± 0.20[a]	0.65 ± 0.23[a]	<0.001

Data are presented as mean ± SD. Comparisons among three groups were performed using one-way ANOVA test followed by Bonferroni's *post hoc* test. Lowercase letter (a) indicates significant difference in comparison with NO HCC-APRI <0.5 group only, (b) indicates significant difference in comparison with NO HCC-APRI≥0.5 only. These comparisons were performed by Student's *t* test.
ALT, alanine aminotransferase; ALP, alkaline phosphatase; APRI, AST-to-platelet ratio index; AST, aspartate aminotransferase; ESR, erythrocyte sedimentation rate; FIB-4, fibrosis-4 index; GFR, glomerular filtration rate; GGT, gamma-glutamyltransferase; HbA1c, glycated haemoglobin; HCC, hepatocellular carcinoma; HDL-c, HDL-cholesterol; hs-CRP, high-sensitivity C-reactive protein; LDL-c, LDL-cholesterol; LM, lymphocyte to monocyte ratio; MH ratio, monocyte to HDL ratio; ML ratio, monocyte to lymphocyte ratio; NL ratio, neutrophil to lymphocyte ratio; TSH, thyroid stimulating hormone; WBC, white blood cell.

Statistical analysis

Descriptive statistical analyses of the study sample were performed, and results were expressed as mean±SD. Comparisons of socio-demographic and clinical variables between two groups were conducted with the *t* test (for continuous variables) and the Pearson $\chi 2$ test (for categorical variables). Statistical analysis between more than two groups was performed by one-way ANOVA followed by Bonferroni *post hoc* test, where required. Correlation between continuous variables was also analysed and estimated using Pearson's correlation coefficient (r). *p* values lower than 0.05 were considered significant. All statistical analyses were performed using the NCSS 12 Statistical Software, version 12.0.2018 (NCSS, LLC Company) and GraphPad Prism, version 9.1.0 (GraphPad Software; San Diego, USA).

Results

Clinical characterisation of the study population

A total of 1,234 participants were enrolled in the present study. All data were generated in an age-adjusted model to minimize age-related significant differences. In order to identify a low-cost and non-invasive predictive factor for HCC, patients were categorized based on APRI score levels.

Among the overall population during the first evaluation, 1,084 patients (498 males and 586 females) had APRI <0.5, which

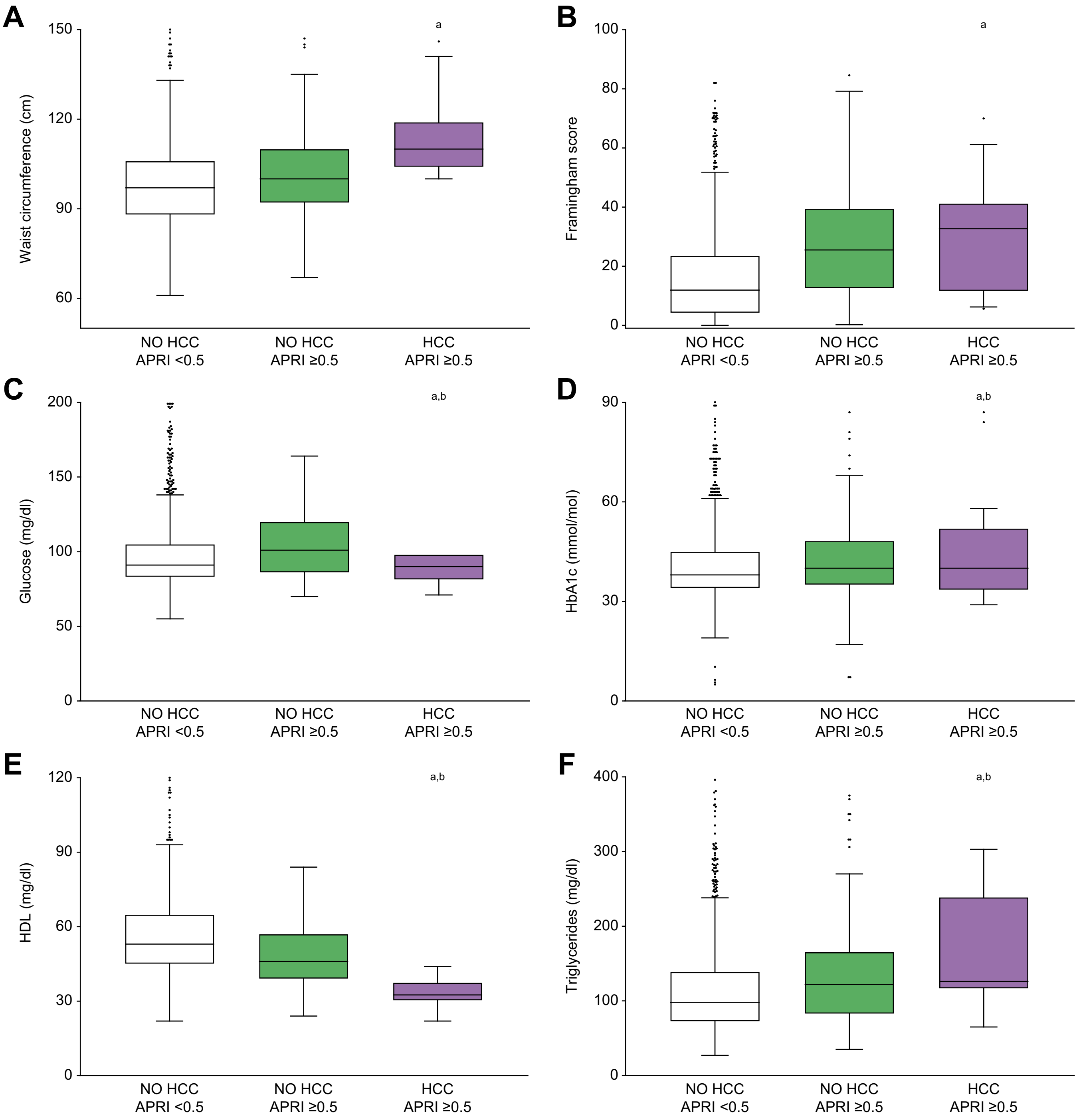

Fig. 1. Metabolic syndrome-associated biomarkers in relation to liver fibrosis and development of HCC. Comparison of metabolic syndrome-associated biomarkers among patients without fibrosis (NO HCC-APRI <0.5) and those with liver fibrosis who will not develop HCC (NO HCC-APRI ≥0.5) or develop HCC (HCC-APRI ≥0.5). The box plots show the median (second quartile), first and third quartile, Tukey whiskers go 1.5 times the interquartile distance or to the highest or lowest point, whichever is shorter. Any data beyond these whiskers are shown as points. Comparisons were performed using one-way ANOVA test followed by Bonferroni's *post hoc* test. Multiple comparison was performed by Student's *t* test. Lowercase letter indicates significant difference with NO HCC-APRI <0.5 (a) and NO HCC-APRI ≥0.5 (b). APRI, AST-to-platelet ratio index; AST, aspartate aminotransferase; HbA1c, glycated haemoglobin; HCC, hepatocellular carcinoma.

rules out the presence of liver fibrosis,[20] and none of them developed liver cancer in the following 5 years (group 1, NO HCC-APRI <0.5). Conversely, a total of 150 patients had APRI ≥0.5: of whom 134 (94 males and 40 females) did not develop liver cancer (group 2, NO HCC-APRI ≥0.5), whereas 16 patients (13 males and 3 females) developed HCC in the following 5 years (group 3, HCC-APRI ≥0.5).

Statistical comparisons among the three groups pointed out that patients in the HCC-APRI ≥0.5 group exhibited increased body weight, BMI, WC, as well as CVR at baseline compared to

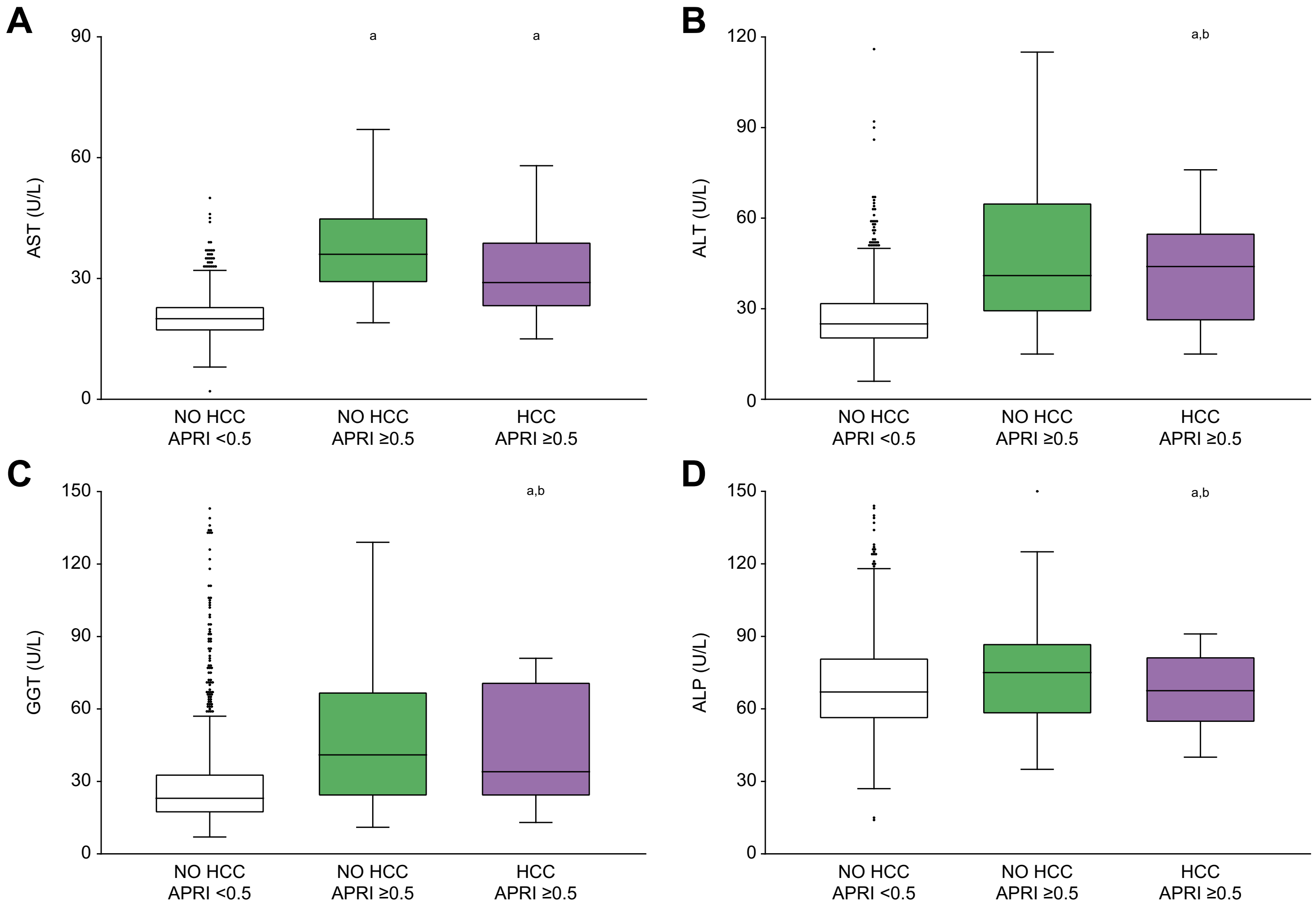

Fig. 2. Transaminase levels in relation to liver fibrosis and development of HCC. Comparison of transaminase levels among patients without fibrosis (NO HCC-APRI <0.5) and those with liver fibrosis who will not develop HCC (NO HCC-APRI ≥0.5) or develop HCC (HCC-APRI ≥0.5). The box plots show the median (second quartile), first and third quartile, Tukey whiskers go 1.5 times the interquartile distance or to the highest or lowest point, whichever is shorter. Any data beyond these whiskers are shown as points. Comparisons were performed using one-way ANOVA test followed by Bonferroni's *post hoc* test. Multiple comparison was performed by Student's *t* test. Lowercase letter indicates significant difference with NO HCC-APRI <0.5 (a) and NO HCC-APRI ≥0.5 (b). ALT, alanine aminotransferase; ALP, alkaline phosphatase; APRI, AST-to-platelet ratio index; AST, aspartate aminotransferase; GGT, gamma-glutamyltransferase; HCC, hepatocellular carcinoma.

those in the NO HCC-APRI <0.5 and NO HCC-APRI ≥0.5 groups. Lower HDL-c and 25-OH vitamin D values were observed in HCC-APRI ≥0.5 patients when compared to the two other groups. No significant differences were found for high-sensitivity C-reactive protein, erythrocyte sedimentation rate, and white blood cell count. Evaluating non-invasive fibrosis scores, the HCC-APRI ≥0.5 group presented significantly higher APRI and FIB-4 scores compared to those who did not have liver fibrosis (Table 1). Focusing on liver function parameters, no difference in albumin level was found among the three groups. Consistent with the absence of cirrhosis in patients who later developed HCC, normal mean values for bilirubin (1.12±0.61 mg/dl) and international normalised ratio (1.10±0.13) were observed in the HCC-APRI ≥0.5 group. Moreover, these patients had low mean Child-Pugh (5.88±0.88) and MELD-Na (8.81±1.70) scores (Table S1).

HCC predicting biomarkers

To better understand the link between visceral obesity and HCC development, we compared WC levels among groups, showing that patients who developed HCC had increased WC at baseline, especially compared to the NO HCC-APRI <0.5 group (Fig. 1A). The analysis of CVR, glucose and glycated haemoglobin (HbA1c) values revealed that these parameters were all increased in the HCC-APRI ≥0.5 group, especially when compared to the NO HCC-APRI ≥0.5 group (Fig. 1B-D).

Given the potential role of HDL-c in HCC pathogenesis,[27] we considered the baseline level of HDL-c in the study population. Intriguingly, we observed that HDL-c was significantly lower at baseline in the HCC-APRI ≥0.5 group, compared to both other groups (Fig. 1E). This could indicate that patients with fibrosis who will develop HCC display a significantly lower HDL-c level. In contrast, triglyceride (TG) levels were significantly higher in the HCC-APRI ≥0.5 group (Fig. 1F).

Transaminases and liver fibrosis scores[28] may be predictive of liver cancer development, therefore, we further analysed AST, ALT, gamma-glutamyltransferase (GGT) and alkaline phosphatase (ALP) levels, which were all significantly higher in the HCC-APRI ≥0.5 group (Fig. 2A-D). In particular, AST levels were higher also in the NO HCC-APRI ≥0.5 group, whereas the more liver-specific markers ALT and GGT were significantly higher in the

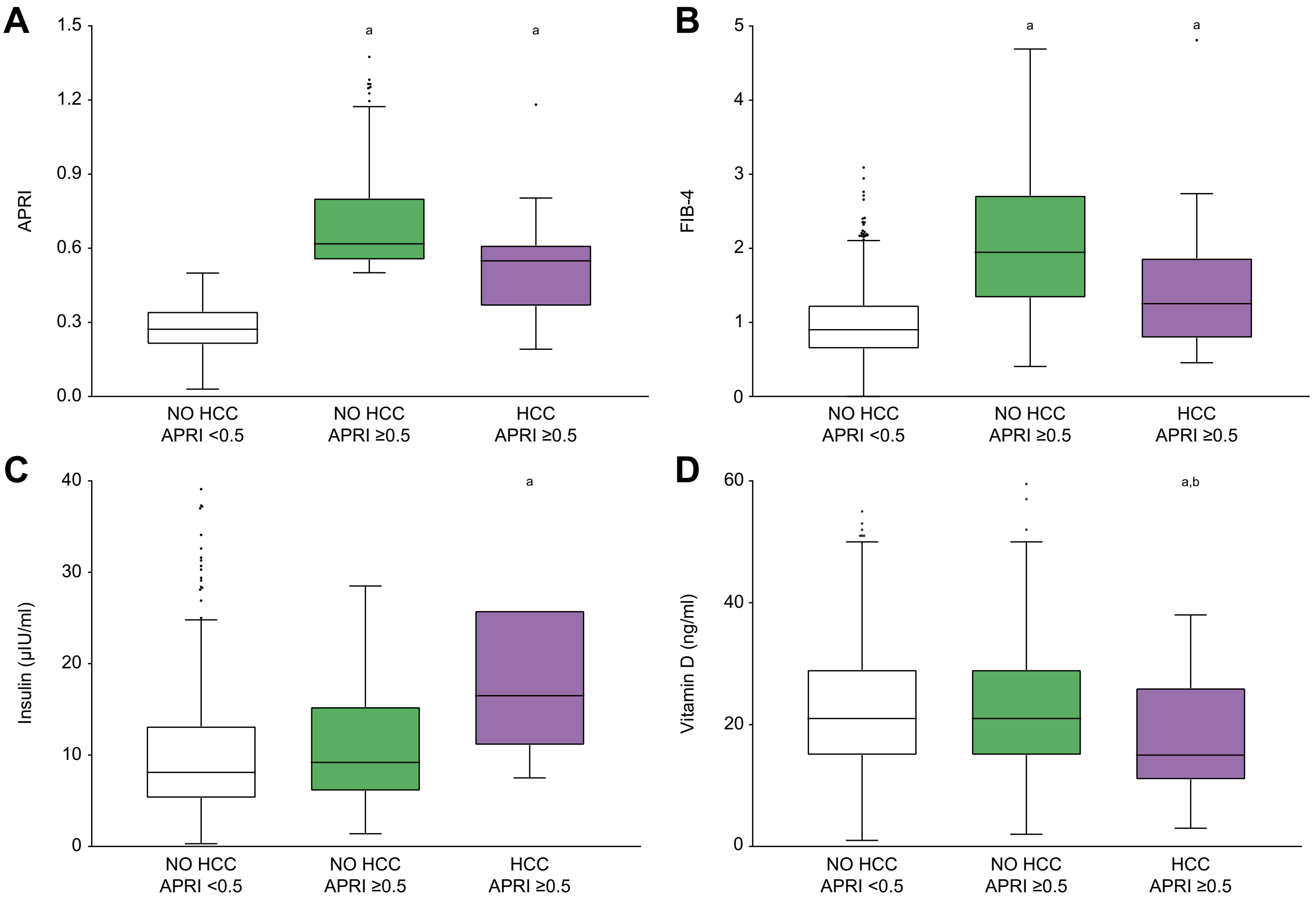

Fig. 3. Non-invasive liver fibrosis scores, insulin, and vitamin D levels in relation to liver fibrosis and development of HCC. Comparison among patients without fibrosis (NO HCC-APRI <0.5) and those with liver fibrosis who will not develop HCC (NO HCC-APRI ≥0.5) or develop HCC (HCC-APRI ≥0.5). The box plots show the median (second quartile), first and third quartile, Tukey whiskers go 1.5 times the interquartile distance or to the highest or lowest point, whichever is shorter. Any data beyond these whiskers are shown as points. Comparisons were performed using one-way ANOVA test followed by Bonferroni's *post hoc* test. Multiple comparison was performed by Student's *t* test. Lowercase letter indicates significant difference with NO HCC-APRI <0.5 (a) and NO HCC-APRI ≥0.5 (b). APRI, AST-to-platelet ratio index; AST, aspartate aminotransferase; FIB-4, fibrosis-4; HCC, hepatocellular carcinoma.

HCC-APRI ≥0.5 group, even when compared to the NO HCC-APRI ≥0.5 group. Therefore, since transaminases are used to determine liver fibrosis scores, it is not surprising that APRI and FIB-4 indexes were higher in the HCC-APRI ≥0.5 group (Fig. 3A,B).

Impaired blood glucose and insulin sensitivity have been variably associated with HCC,[29] and in our population, patients in the HCC-APRI ≥0.5 group had significantly higher levels of glucose and HbA1c compared to the other two groups. Moreover, a significant difference in insulin levels was observed, particularly between NO HCC-APRI <0.5 and HCC-APRI ≥0.5 groups (Fig. 3C). Finally, to better understand the potential role of vitamin D in liver cancer prediction,[30] levels of 25-OH vitamin D were analysed; we observed significantly lower baseline values in patients who later developed HCC (Fig. 3D).

HCC prognostic factors in liver fibrosis

Alterations in WC, HDL-c, TG, glucose, and 25-OH vitamin D levels have frequently been associated with HCC, and data presented in this study confirms these associations. To further study the relevance of these observations, the correlation between these variables in NO HCC-APRI ≥0.5 and HCC-APRI ≥0.5 groups were evaluated. A strong negative correlation between WC and HDL-c level was detected in the HCC-APRI ≥0.5 group ($r = 0.93$, $p <0.01$) but not in the NO HCC-APRI ≥0.5 group ($r = 0.27$, $p =$ n.s.) (Fig. 4A). Similarly, the correlation between increased WC parameters and high TG levels is stronger in the HCC-APRI ≥0.5 group ($r = 0.8$, $p <0.01$) than in the NO HCC-APRI ≥0.5 group ($r = 0.31$; $p <0.05$) (Fig. 4B). Analysis of the association between glucose level and WC revealed a stronger significant correlation in the HCC-APRI ≥0.5 ($r = 0.63$, $p <0.05$) than in the NO HCC-APRI ≥0.5 group (Fig. 4C). Finally, a linear regression analysis between 25-OH vitamin D and HDL was performed in the NO HCC-APRI ≥0.5 and HCC-APRI ≥0.5 groups, revealing a significant negative correlation only in the latter group (Fig. 4D). In particular, patients in the HCC-APRI ≥0.5 group displayed low HDL-c (<45 mg/dl) with a corresponding low level of vitamin D (<20 ng/ml), confirming that patients developing HCC are characterised by concomitant lower levels of both HDL-c and vitamin D.

HCC prognostic factors in patients with low HDL-c

Dot plot representation of HDL values in the three groups showed that there are a significant number of patients who, despite not being affected by HCC, exhibited lower HDL-c, similar to those in the HCC-APRI ≥0.5 group (Fig. 5A). To

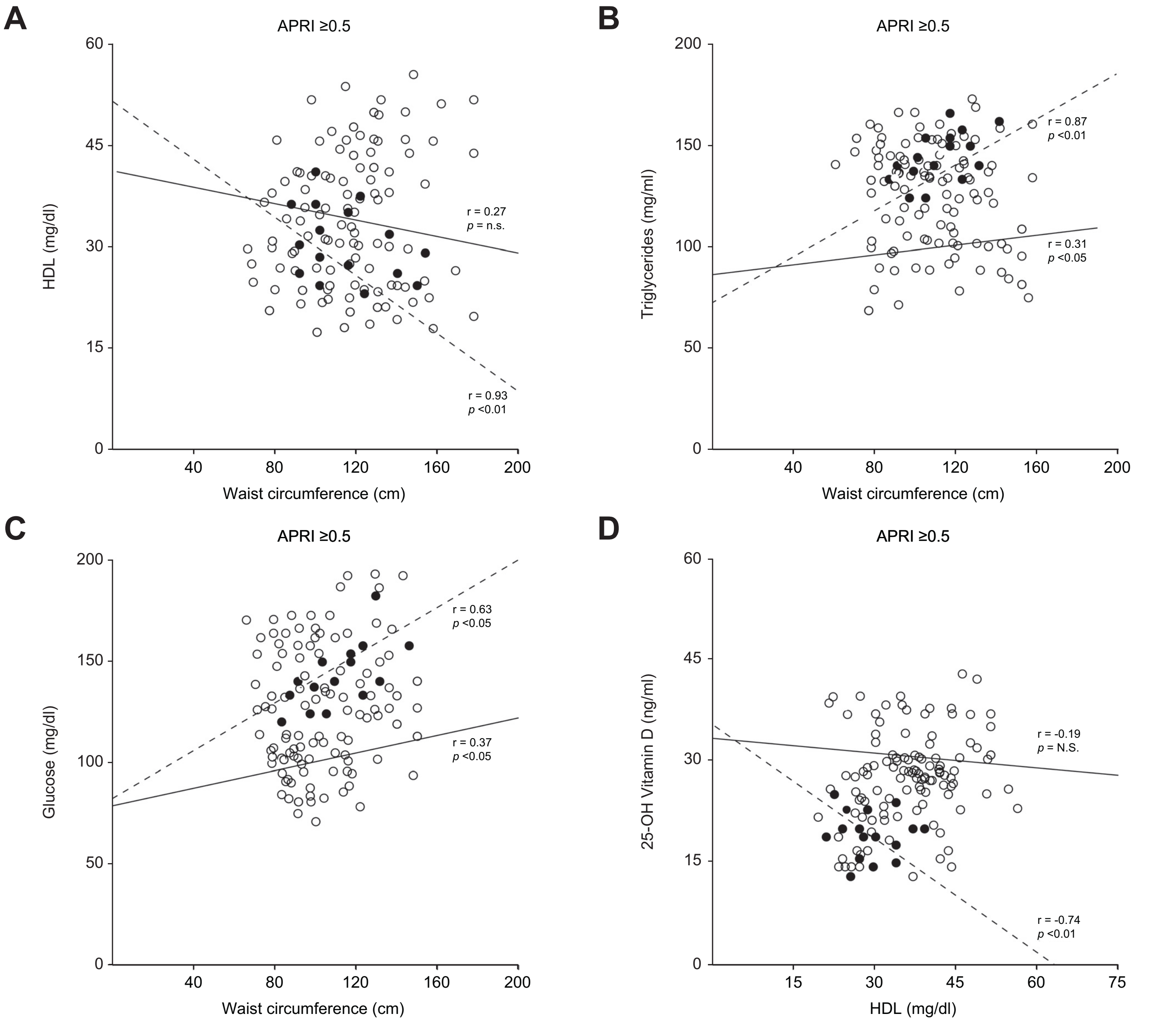

Fig. 4. Relationships of waist circumference with metabolic biomarker and of HDL-c with vitamin D level in patients with APRI ≥0.5. Dashed line and black circles represent HCC patients, while continuous line and white circles represent NO HCC patients. r indicates Pearson's correlation coefficient; *p* indicates statistical significance. APRI, AST-to-platelet ratio index; AST, aspartate aminotransferase; HDL-c, HDL-cholesterol.

explain why, *ceteris paribus*, some patients with low HDL developed cancer, the reported maximum value of HDL-c (50.00 mg/dl) in the HCC-APRI ≥0.5 group was set as a cut-off value for the overall population, and then biochemical and anthropometric variables which had shown a significant difference in the first analysis were further analysed in 484 patients with HDL-c ≤50.00 mg/dl (374 belonging to the first group, 94 belonging to the second one, 16 developing HCC). Multiple comparisons among the three groups were performed, in an age-adjusted model (Table 2). The statistical significance of higher GGT value was present when comparing both NO HCC-APRI ≥0.5 and HCC-APRI ≥0.5 groups to the NO HCC-APRI <0.5 group, so does not reliably characterise patients with HCC among those with HDL-c <50 mg/dl and liver fibrosis (Fig. 5B). 25-OH vitamin D remained significantly decreased in the HCC-APRI ≥0.5 group (Fig. 5C). Similarly, although BMI and body weight as well as glycemia retained a positive trend, they lost their statistical power among patients with lower HDL. Conversely, higher HbA1c and WC, strongly maintained their statistical significance (p <0.05) (Fig. 5D,E).

Discussion

In this study, low HDL-c levels were associated with an increased risk of developing HCC, thus representing an important discriminating factor to predict the onset of HCC among patients

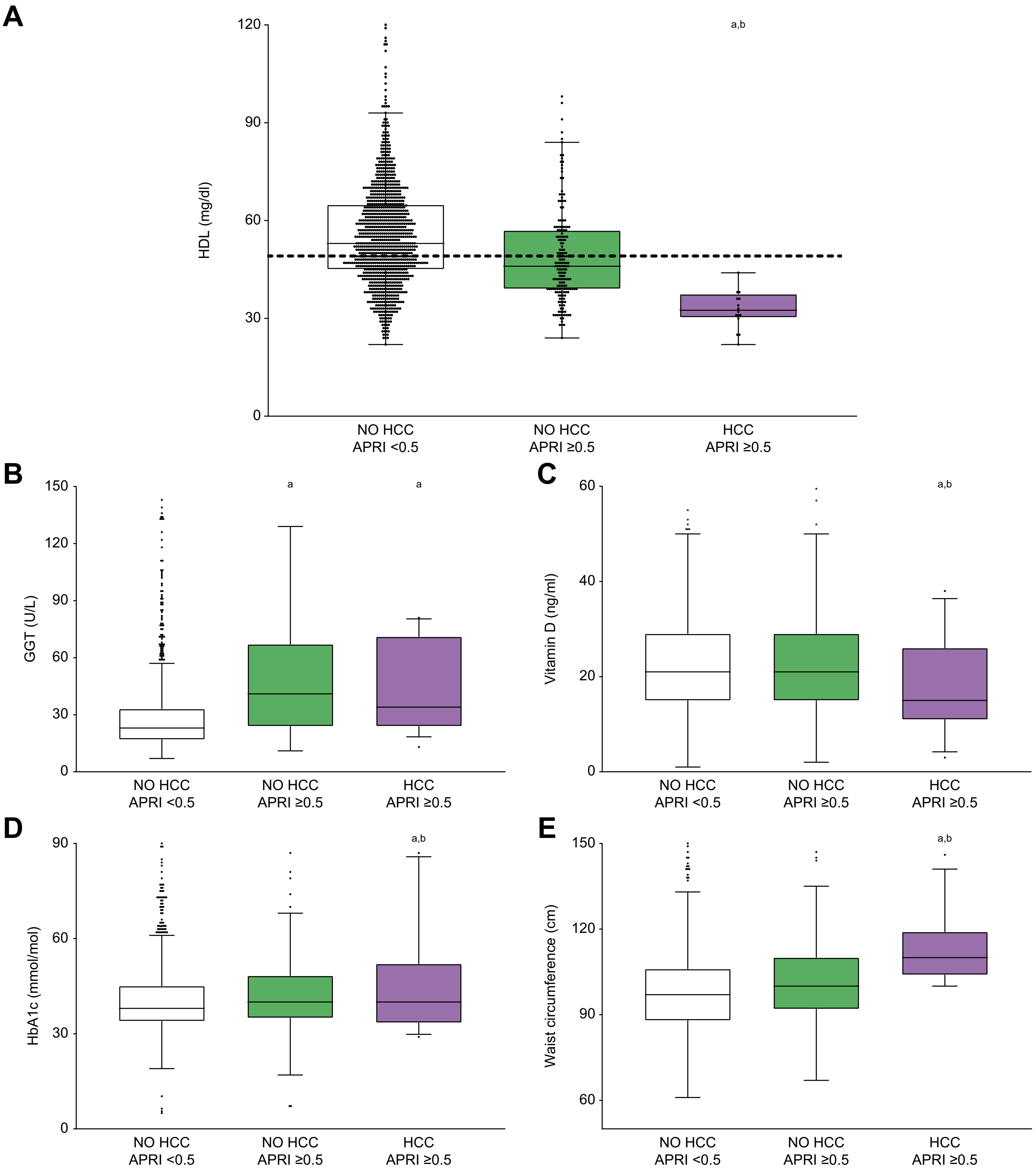

Fig. 5. Comparison among patients with HDL-c ≤50 mg/dl. (A) Dot and box plots representation of HDL-c in patients without fibrosis (NO HCC-APRI <0.5) and those with liver fibrosis who will not develop HCC (NO HCC-APRI ≥0.5) or develop HCC (HCC-APRI ≥0.5). Dashed line shows the maximum HDL-c value in HCC-APRI ≥0.5 group, that is 50 mg/dl. Comparison of GGT level (B), 25-OH vitamin D (C), HbA1c (D), and WC (E) in these three groups among patients with HDL-c ≤50 mg/dl. The box plots show the median (second quartile), first and third quartile, Tukey whiskers go 1.5 times the interquartile distance or to the highest or lowest point, whichever is shorter. Any data beyond these whiskers are shown as points. Comparisons were performed using one-way ANOVA test followed by Bonferroni's *post hoc* test. Multiple comparison was performed by Student's *t* test. Lowercase letter indicates significant difference with NO HCC-APRI <0.5 (a) and NO HCC-APRI ≥0.5 (b). APRI, AST-to-platelet ratio index; AST, aspartate aminotransferase; GGT, gamma-glutamyltransferase; HbA1c, glycated haemoglobin; HCC, hepatocellular carcinoma.

Table 2. Clinical characterisation of the study population with HDL-c ≤50 mg/dl.

	NO HCC-APRI <0.5	NO HCC-APRI ≥0.5	HCC-APRI ≥0.5	
	374 M:F (229:145)	94 M:F (62:32)	16 M:F (13:3)	
Clinical variable	Mean ± SD	Mean ± SD	Mean ± SD	*p* value
Weight (kg)	82.01 ± 16.01	83.77 ± 17.58	96.07 ± 19.46	n.s.
BMI (kg/m^2)	29.16 ± 5.93	29.61 ± 5.75	32.59 ± 6.89	n.s.
Waist circumference (cm)	98.83 ± 14.04	103.52 ± 14.10	114.30 ± 11.82[a,b]	<0.05
Cardiovascular risk (Framingham)	22.23 ± 18.18	34.30 ± 21.97[a]	34.74 ± 18.98[a]	<0.05
HDL-c (mg/dl)	42.55 ± 6.23	39.87 ± 6.16	36.72 ± 8.31[a,b]	<0.05
Triglycerides (mg/dl)	175.08 ± 45.60	179.13 ± 43.58	184.4 ± 45.92	n.s.
Glucose (mg/dl)	109.62 ± 39.18	108.59 ± 29-02	131.3 ± 65.21	n.s.
HbA1c (mmol/mol)	44.44 ± 15.16	42.39 ± 12.24	53.11 ± 19.31[a,b]	<0.05
25-OH vitamin D (ng/ml)	19.48 ± 9.15	20.76 ± 9.83	17.73 ± 7.26[a,b]	<0.05
hs-CRP (mg/L)	4.94 ± 4.99	3.77 ± 2.34	3.93 ± 2.08	n.s.
ESR (mm/h)	17.76 ± 15.27	19.88 ± 19.03	18.83 ± 16.51	n.s.
GFR (ml/min)	99.23 ± 2.43	91.77 ± 1.48[a]	86.41 ± 6.20[a]	<0.05
Platelet count (10^3/μl)	246.86 ± 65.03	180.69 ± 46.68[a]	206.7 ± 49.86[a]	<0.05
GGT (U/L)	34.30 ± 23.66	55.35 ± 33.58[a]	51 ± 25.02[a]	<0.05
AST (U/L)	20.50 ± 5.81	41.23 ± 22.14[a]	36.6 ± 8.93[a]	<0.05
ALT (U/L)	29.45 ± 11.67	56.93 ± 33.98[a]	47 ± 18.16[a,b]	<0.05
ALP (U/L)	73.36 ± 22.61	75.58 ± 22.60	70.62 ± 20.13	n.s.
Albumin (g/dl)	4.46 ± 0.04	4.42 ± 0.06	4.46 ± 0.27	n.s.
MH ratio	10.93 ± 4.44	10.21 ± 3.81	10.21 ± 3.81	n.s.
FIB-4 index	0.95 ± 0.45	2.11 ± 1.18[a]	1.52 ± 1.25[a,b]	<0.05
APRI score	0.28 ± 0.01	0.74 ± 0.34[a]	0.54 ± 0.26[a]	<0.05

Data is presented as mean ± SD. Comparisons among three groups were performed using one-way ANOVA test followed by Bonferroni's *post hoc* test. Lowercase letter (a) indicates significant difference in comparison with NO HCC-APRI <0.5 group only, (b) indicates significant difference in comparison with NO HCC-APRI ≥0.5 only. These comparisons were performed by Student's *t* test.
ALT, alanine aminotransferase; ALP, alkaline phosphatase; APRI, AST-to-platelet ratio index; AST, aspartate aminotransferase; ESR, erythrocyte sedimentation rate; FIB-4, fibrosis-4; GFR, glomerular filtration rate; GGT, gamma-glutamyltransferase; HCC, hepatocellular carcinoma; HDL-c, HDL-cholesterol; hs-CRP, high-sensitivity C-reactive protein; LDL-c, LDL-cholesterol; MH ratio, monocyte to HDL ratio.

without cirrhosis but with liver fibrosis, one of the clinical manifestations of NAFLD. From a clinical perspective, the early identification of patients with fibrosis presenting with a higher risk of progression towards severe forms of liver disease, including HCC, is crucial.

Although the degree of fibrosis is the strongest predictive factor for liver-related and all-cause mortality, the causative factors for NAFLD progression towards fibrosis are still not known[31] and HDL-c levels have also been proposed to predict decompensation in patients with chronic liver disease[32] and are associated with a more aggressive phenotype,[33] recurrence after curative resections,[34] and worse outcomes in patients with HCC.[35]

Investigating whether the relationship between HDL-c and cancer incidence is causative, Pirro *et al.* concluded that several HDL pathway's components are crucially connected with cancer cell proliferation and survival, speculating that impaired RCT may contribute to cancer onset and progression.[36] Hepatic cancer cells display a higher receptor-mediated uptake of HDL than normal cells, thus potentially explaining the low plasma HDL-c level found in patients with HCC.[35] Moreover, alterations of liver X receptors (LXRs), the master regulator of cholesterol homeostasis and RCT, are involved in the progression of HCC.[37] Indeed, in physiological conditions, increased amounts of the cholesterol by-products oxysterols activate LXRs and promote the expression of their target genes, keeping cholesterol level within a specific range inside the cell and intensifying the production of HDL in the liver, adipose tissue, adrenal glands, intestine, and macrophages. However, in rapidly growing cells, characterised by a high-energy demand (just as in cancer cells) a paradoxical suppression of LXR-driven pathways has been detected, suggesting a possible uncoupling between the high cholesterol concentration needed to sustain active proliferation and LXR activation.[38,39]

In our population, around 30% of patients who did not develop HCC had low levels of HDL, suggesting that higher HDL-c levels might protect against HCC and that conversely, in patients with lower HDL-c, some additional metabolic factors may drive disease onset. To this end, we found HbA1c and WC were significantly increased in patients who developed HCC having presented with low HDL-c at time 0. The role of WC, but not BMI, in predicting HCC among patients with lower HDL-c highlights one more time the importance of assessing abdominal fat in clinical evaluation and supports the concept that visceral adiposity and associated conditions, such as low-grade inflammation, adipokines release, and insulin resistance, may play a pivotal role in carcinogenesis.[40] Consequently, those conditions leading to fat accumulation, such as high-calorie intake and unbalanced lifestyles, could boost HCC development in patients at high risk, mediated by low HDL-c. Accumulation of hepatic lipids, due to altered metabolism or dietary choices (including high-carbohydrate, high-fat diets), favours the production of potentially toxic metabolites, which damage the liver and lead to increased hepatic scarring.[41,42] Subsequent progressive inflammation and, eventually, chronic necroinflammation and fibrosis, compensatory proliferation, and a chronic regenerative environment contribute to HCC development.[43,44] Also, preclinical models have highlighted the impact of dietary choices and excessive caloric-intake on cancer onset and development.[45,46] In this context, it has been shown that dietary cholesterol can also modulate the intestinal microbiota, contributing to the sequential progression of steatosis to steatohepatitis, fibrosis and finally HCC in mice.[47]

Furthermore, when analysing the subpopulation presenting with low HDL-c, we found that reduced 25-OH vitamin D level was still associated with HCC, as already detected in the first analysis. At a molecular level, this liposoluble hormone precursor is transformed into 1,25-OH vitamin D which is the active hormone form that binds to the vitamin D receptor (VDR), a metabolic nuclear receptor, similar to LXRs. VDR controls expression of genes involved in bile acid synthesis from cholesterol, calcium metabolism, cell differentiation, apoptosis, and immunity.[48] Hepatocytes do not express VDR, while hepatic stellate cells do; therefore, one could speculate that hypovitaminosis D could negatively influence the hepatic inflammatory microenvironment, ultimately laying the ground for hepatic tumorigenesis. After all, chronic low-grade inflammation caused by visceral adiposopathy is another MetS feature that has been proposed to explain higher cancer incidence in individuals who are obese. In a previous study, the combination of elevated iron and low HDL-c plasma levels at baseline was reported to predict cancer risk over almost 15 years.[49] However, in our study, no significant differences in iron levels were detected at baseline.

In conclusion, our data indicates that low HDL levels together with adiposopathy and its associated biomarkers may be considered as useful variables in defining and validating new non-invasive prognostic tools to predict HCC development in patients with liver fibrosis. Furthermore, this study provides novel insights into non-invasive prognostic factors, with HDL-c level being a significant predictor of HCC development in high-risk patients with liver fibrosis and metabolic derangement. Although this study does not disclose a molecular mechanism underlying the presented observation, it does provide the rationale for studying cholesterol metabolism in HCC. Finally, from a clinical perspective, our findings recommend reducing adiposopathy, and targeting its associated dysmetabolic conditions, in patients with liver fibrosis and low HDL-c, to revert steatohepatitis and possibly lower HCC risk, by integrating clinical and therapeutic approaches with dietary regimens and healthy lifestyle.

Abbreviations

ALT, alanine aminotransferase; ALP, alkaline phosphatase; APRI, AST-to-platelet ratio index; AST, aspartate aminotransferase; CVR, cardiovascular risk; FA, fatty acid; FIB-4, fibrosis-4; GGT, gamma-glutamyltransferase; HbA1c, glycated haemoglobin; HCC, hepatocellular carcinoma; HDL-c, HDL-cholesterol; LXRs, liver X receptors; MetS, metabolic syndrome; NAFLD, non-alcoholic fatty liver disease; NASH, non-alcoholic steatohepatitis; RCT, reverse cholesterol transport; TG, triglyceride; WC, waist circumference.

Financial support

A.M. is funded by Italian Association for Cancer Research - AIRC IG 2019 Id 23239; EU-JPI HDL-INTIMIC –MIUR FATMAL; MIUR-PON “R&I” 2014–2020 “BIOMIS” cod.ARS01_01220; POR Puglia FESR—FSE 2014– 2020, “INNOMA” cod. 4TCJLV4. E.P. is funded by PON AIM1853334—Attività 2, linea 1.

Conflict of interest

The authors declare no conflict of interest.

Please refer to the accompanying ICMJE disclosure forms for further details.

Authors’ contributions

Conceptualization, A.M.; methodology-visualization, L.C. and C.D.M.; software-formal analysis, C.D.M.; investigation, L.C., C.D.M., and E.D.B.; resources, G.P. and P.S.; data curation, L.C., C.D.M, E.D.B.; writing—original draft preparation, L.C., R.M.G., M.C. and E.P.; writing—review and editing, E.P., A.M.; supervision, C.S., E.P., and A.M.; project administration, A.M.; funding acquisition, A.M. All authors have read and agreed to the published version of the manuscript.

Data availability statement

The data that supports the findings of this study is available from the corresponding author upon reasonable request.

Acknowledgements

We thank the physicians and nurses of the Unità Operativa Complessa Universitaria di Medicina Interna “Cesare Frugoni” of the Azienda Ospedaliero—Universitaria Policlinico di Bari for their help and support during the study. A special thanks to Roberta Le Donne and Antonella De Ruvo for their support.

Supplementary data

Supplementary data to this article can be found online at https://doi.org/10.1016/j.jhepr.2022.100627.

References

[1] Forner A, Reig M, Bruix J. Hepatocellular carcinoma. The Lancet 2018;391:1301–1314.
[2] Calle EE, Rodriguez C, Walker-Thurmond K, Thun MJ. Overweight, obesity, and mortality from cancer in a prospectively studied cohort of U.S. adults. N Engl J Med 2003;348:1625–1638.
[3] McGlynn KA, Petrick JL, El-Serag HB. Epidemiology of hepatocellular carcinoma. Hepatology 2021;73:4–13.
[4] Chalasani N, Younossi Z, Lavine JE, Charlton M, Cusi K, Rinella M, et al. The diagnosis and management of nonalcoholic fatty liver disease: practice guidance from the American Association for the Study of Liver Diseases. Hepatology 2018;67:328–357.
[5] Nderitu P, Bosco C, Garmo H, Holmberg L, Malmström H, Hammar N, et al. The association between individual metabolic syndrome components, primary liver cancer and cirrhosis: a study in the Swedish AMORIS cohort: association between individual MetS components, PLC and Cirrhosis. Int J Cancer 2017;141:1148–1160.
[6] Piccinin E, Villani G, Moschetta A. Metabolic aspects in NAFLD, NASH and hepatocellular carcinoma: the role of PGC1 coactivators. Nat Rev Gastroenterol Hepatol 2019;16:160–174.
[7] Mittal S, El-Serag HB, Sada YH, Kanwal F, Duan Z, Temple S, et al. Hepatocellular carcinoma in the absence of cirrhosis in United States veterans is associated with nonalcoholic fatty liver disease. Clin Gastroenterol Hepatol 2016;14:124–131.e1.
[8] Hanahan D, Weinberg RA. Hallmarks of cancer: the next generation. Cell 2011;144:646–674.
[9] Rudalska R, Harbig J, Snaebjornsson MT, Klotz S, Zwirner S, Taranets L, et al. LXRα activation and Raf inhibition trigger lethal lipotoxicity in liver cancer. Nat Cancer 2021;2:201–217.
[10] Menendez JA, Lupu R. Fatty acid synthase and the lipogenic phenotype in cancer pathogenesis. Nat Rev Cancer 2007;7:763–777.
[11] Xie Y, Sun R, Gao L, Guan J, Wang J, Bell A, et al. Chronic activation of LXRα sensitizes mice to hepatocellular carcinoma. Hepatol Commun 2022;6:1123–1139.
[12] Reed J, Lardy HA. Mode of action of hypoglycemic agents. 3. Studies on 5-methoxy indole-2-carboxylic acid and quinaldic acid. J Biol Chem 1970;245:5297–5303.
[13] Piccinin E, Peres C, Bellafante E, Ducheix S, Pinto C, Villani G, et al. Hepatic peroxisome proliferator-activated receptor γ coactivator 1β drives mitochondrial and anabolic signatures that contribute to hepatocellular carcinoma progression in mice. Hepatology 2018;67:884–898.
[14] Triki M, Rinaldi G, Planque M, Broekaert D, Winkelkotte AM, Maier CR, et al. mTOR signaling and SREBP activity increase FADS2 expression and can activate sapienate biosynthesis. Cell Rep 2020;31:107806.
[15] Marra F, Svegliati-Baroni G. Lipotoxicity and the gut-liver axis in NASH pathogenesis. J Hepatol 2018;68:280–295.
[16] Saponaro C, Sabatini S, Gaggini M, Carli F, Rosso C, Positano V, et al. Adipose tissue dysfunction and visceral fat are associated with hepatic

insulin resistance and severity of NASH even in lean individuals. Liver International 2022;42:2418–2427. https://doi.org/10.1111/liv.15377.
[17] Mackness MI, Durrington PN. HDL, its enzymes and its potential to influence lipid peroxidation. Atherosclerosis 1995;115:243–253.
[18] Ahn J, Lim U, Weinstein SJ, Schatzkin A, Hayes RB, Virtamo J, et al. Prediagnostic total and high-density lipoprotein cholesterol and risk of cancer. Cancer Epidemiol Biomarkers Prev 2009;18:2814–2821.
[19] Härdfeldt J, Cariello M, Simonelli S, Ossoli A, Scialpi N, Piglionica M, et al. Abdominal obesity negatively influences key metrics of reverse cholesterol transport. Biochim Biophys Acta Mol Cel Biol Lipids 2022;1867: 159087.
[20] Zhong G-C, Huang S-Q, Peng Y, Wan L, Wu Y-Q-L, Hu T-Y, et al. HDL-C is associated with mortality from all causes, cardiovascular disease and cancer in a J-shaped dose-response fashion: a pooled analysis of 37 prospective cohort studies. Eur J Prev Cardiolog 2020;27:1187–1203.
[21] Benn M, Tybjærg-Hansen A, Smith GD, Nordestgaard BG. High body mass index and cancer risk—a Mendelian randomisation study. Eur J Epidemiol 2016;31:879–892.
[22] Zhao W, Guan J, Horswell R, Li W, Wang Y, Wu X, et al. HDL cholesterol and cancer risk among patients with type 2 diabetes. Diabetes Care 2014;37:3196–3203.
[23] De Matteis C, Cariello M, Graziano G, Battaglia S, Suppressa P, Piazzolla G, et al. AST to Platelet Ratio Index (APRI) is an easy-to-use predictor score for cardiovascular risk in metabolic subjects. Sci Rep 2021;11:14834.
[24] Pugh RN, Murray-Lyon IM, Dawson JL, Pietroni MC, Williams R. Transection of the oesophagus for bleeding oesophageal varices. Br J Surg 1973;60:646–649.
[25] Kim WR, Biggins SW, Kremers WK, Wiesner RH, Kamath PS, Benson JT, et al. Hyponatremia and mortality among patients on the liver-transplant waiting list. N Engl J Med 2008;359:1018–1026.
[26] meld_na [Internet]. Available from: https://optn.transplant.hrsa.gov/media/1575/policynotice_20151101.pdf.
[27] Zhao L, Deng C, Lin Z, Giovannucci E, Zhang X. Dietary fats, serum cholesterol and liver cancer risk: a systematic review and meta-analysis of prospective studies. Cancers (Basel) 2021;13:1580.
[28] Sun M, Wang W, Liu X, Wang Y, Cui H, Liu S, et al. Total cholesterol, alanine aminotransferase and the risk of primary liver cancer: a population-based prospective study. Medicine (Baltimore) 2021;100: e25746.
[29] Han H, Zhang T, Jin Z, Guo H, Wei X, Liu Y, et al. Blood glucose concentration and risk of liver cancer: systematic review and meta-analysis of prospective studies. Oncotarget 2017;8:50164–50173.
[30] Yi Z, Wang L, Tu X. Effect of vitamin D deficiency on liver cancer risk: a systematic review and meta-analysis. Asian Pac J Cancer Prev 2021;22:991–997.
[31] Desterke C, Chiappini F. Lipid related genes altered in NASH connect inflammation in liver pathogenesis progression to HCC: a canonical pathway. Int J Mol Sci 2019;20:E5594.
[32] Rao BH, Nair P, Koshy AK, Krishnapriya S, Greeshma CR, Venu RP. Role of high-density lipoprotein cholesterol (HDL-C) as a clinical predictor of decompensation in patients with chronic liver disease (CLD). Int J Hepatol 2021;2021:1795851.
[33] Akkiz H, Carr BI, Guerra V, Donghia R, Yalçın K, Karaoğullarından U, et al. Plasma lipids, tumor parameters and survival in HCC patients with HBV and HCV. J Transl Sci 2021;7.
[34] Tian L, Yu Q, Gao X-H, Wu J, Ma X-L, Dai Q, et al. A new use for an old index: preoperative high-density lipoprotein predicts recurrence in patients with hepatocellular carcinoma after curative resections. Lipids Health Dis 2017;16:123.
[35] Jiang S-S, Weng D-S, Jiang L, Zhang Y-J, Pan K, Pan Q-Z, et al. The clinical significance of preoperative serum cholesterol and high-density lipoprotein-cholesterol levels in hepatocellular carcinoma. J Cancer 2016;7:626–632.
[36] Pirro M, Ricciuti B, Rader DJ, Catapano AL, Sahebkar A, Banach M. High density lipoprotein cholesterol and cancer: marker or causative? Prog Lipid Res 2018;71:54–69.
[37] Long H, Guo X, Qiao S, Huang Q. Tumor LXR expression is a prognostic marker for patients with hepatocellular carcinoma. Pathol Oncol Res 2018;24:339–344.
[38] Lo Sasso G, Celli N, Caboni M, Murzilli S, Salvatore L, Morgano A, et al. Down-regulation of the LXR transcriptome provides the requisite cholesterol levels to proliferating hepatocytes. Hepatology 2010;51:1334–1344.
[39] Lo Sasso G, Bovenga F, Murzilli S, Salvatore L, Di Tullio G, Martelli N, et al. Liver X receptors inhibit proliferation of human colorectal cancer cells and growth of intestinal tumors in mice. Gastroenterology 2013;144:1497–1507.e13.
[40] Crudele L, Piccinin E, Moschetta A. Visceral adiposity and cancer: role in pathogenesis and prognosis. Nutrients 2021;13:2101.
[41] Masuzaki R, Karp SJ, Omata M. NAFLD as a risk factor for HCC: new rules of engagement? Hepatol Int 2016;10:533–534.
[42] Bovenga F, Sabbà C, Moschetta A. Uncoupling nuclear receptor LXR and cholesterol metabolism in cancer. Cell Metab 2015;21:517–526.
[43] Anstee QM, Reeves HL, Kotsiliti E, Govaere O, Heikenwalder M. From NASH to HCC: current concepts and future challenges. Nat Rev Gastroenterol Hepatol 2019;16:411–428.
[44] Pascual G, Avgustinova A, Mejetta S, Martín M, Castellanos A, Attolini CS-O, et al. Targeting metastasis-initiating cells through the fatty acid receptor CD36. Nature 2017;541:41–45.
[45] Chen M, Zhang J, Sampieri K, Clohessy JG, Mendez L, Gonzalez-Billalabeitia E, et al. An aberrant SREBP-dependent lipogenic program promotes metastatic prostate cancer. Nat Genet 2018;50:206–218.
[46] Ioannou GN, Lee SP, Linsley PS, Gersuk V, Yeh MM, Chen Y, et al. *Pcsk9* deletion promotes murine nonalcoholic steatohepatitis and hepatic carcinogenesis: role of cholesterol. Hepatol Commun 2022;6:780–794.
[47] Zhang X, Coker OO, Chu ES, Fu K, Lau HCH, Wang Y-X, et al. Dietary cholesterol drives fatty liver-associated liver cancer by modulating gut microbiota and metabolites. Gut 2021;70:761–774.
[48] El-Sharkawy A, Malki A. Vitamin D signaling in inflammation and cancer: molecular mechanisms and therapeutic implications. Molecules 2020;25:E3219.
[49] Mainous AG, Wells BJ, Koopman RJ, Everett CJ, Gill JM. Iron, lipids, and risk of cancer in the Framingham offspring cohort. Am J Epidemiol 2005;161:1115–1122.

Journal of Hepatology, Volume *5*

Supplemental information

Low HDL-cholesterol levels predict hepatocellular carcinoma development in individuals with liver fibrosis

Lucilla Crudele, Carlo De Matteis, Elena Piccinin, Raffaella Maria Gadaleta, Marica Cariello, Ersilia Di Buduo, Giuseppina Piazzolla, Patrizia Suppressa, Elsa Berardi, Carlo Sabbà, and Antonio Moschetta

Low HDL-cholesterol levels predict hepatocellular carcinoma development in individuals with liver fibrosis

Lucilla Crudele, Carlo De Matteis, Elena Piccinin, Raffaella Maria Gadaleta, Marica Cariello, Ersilia Di Buduo, Giuseppina Piazzolla, Patrizia Suppressa, Elsa Berardi, Carlo Sabbà, Antonio Moschetta

Table of content

Table S1...2

Table S1. Child-Pugh and Meld Scores in patients who developed HCC.

Patient ID	Child-Pugh	Meld Score-Na
1	6	13
2	5	8
3	6	9
4	5	7
5	6	11
6	8	10
7	5	9
8	5	9
9	5	9
10	5	7
11	6	11
12	6	9
13	7	7
14	7	8
15	6	7
16	5	7
**Mean±SD**	**5.81±0.88**	**8.81±1.70**
